# LncRNA HOXA‐AS2 Can Predict the Risk of Acute Respiratory Distress Syndrome and 28‐Day Mortality in Patients With Sepsis

**DOI:** 10.1111/crj.70082

**Published:** 2025-05-21

**Authors:** Youhong Quan, Song Gao

**Affiliations:** ^1^ Intensive Care Unit Wuxi Branch of Zhongda Hospital Southeast University Wuxi China

**Keywords:** ARDS, diagnosis, HOXA‐AS2, prognosis, sepsis

## Abstract

**Objective:**

This study aimed to explore the diagnostic and predictive value of lncRNA HOXA‐AS2 for acute respiratory distress syndrome (ARDS) and 28‐day mortality in sepsis patients.

**Methods:**

The levels of HOXA‐AS2 in sepsis and ARDS patients were detected by real‐time quantitative reverse transcription PCR (RT‐qPCR). The receiver operating curve (ROC) curve was used to evaluate the diagnostic value of HOXA‐AS2 for sepsis and ARDS. The K‐M curve was used to evaluate the effect of HOXA‐AS2 on the prognosis. Logistic regression analysis and COX regression analysis were used to explore the risk factors influencing ARDS and death. Additionally, an ARDS cell model was constructed to explore the effects of HOXA‐AS2 on cell viability, inflammation, and endothelial glycocalyx.

**Results:**

HOXA‐AS2 decreased in sepsis patients who developed ARDS and died. This molecule can not only serve as a diagnostic marker for sepsis but also act as a risk factor to predict the risk of ARDS and death within 28 days in patients with sepsis. Sepsis patients with low levels of HOXA‐AS2 are more prone to ARDS and death. In cells attacked by lipopolysaccharide (LPS), overexpression of HOXA‐AS2 inhibited apoptosis, inflammation, and the degradation of endothelial glycocalyx.

**Conclusion:**

In sepsis patients, HOXA‐AS2 has the potential to serve as a predictive marker for ARDS and 28‐day mortality. This molecule may delay the progression of ARDS by inhibiting inflammation and the degradation of the endothelial glycocalyx.

## Graphical abstract

LncRNA HOXA‐AS2 is not only a diagnostic marker for sepsis but also can predict the risk of acute respiratory distress syndrome (ARDS) and 28‐day mortality in patients with sepsis.

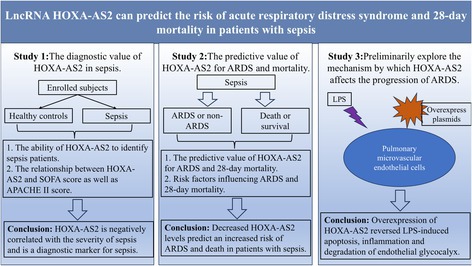

## 1 Introduction

Sepsis is a syndrome characterized by a systemic inflammatory response, which can lead to a dysregulation reaction to bacterial, fungal, viral, and parasitic infections in a patient's body. This can result in multi‐organ failure and, in severe cases, may be life‐threatening [1, 2]. Sepsis continues to be one of the main contributors to the global mortality [3]. Acute respiratory distress syndrome (ARDS) is an acute inflammatory injury and the most common and severe complication in sepsis [4, 5]. Globally, ARDS affects at least seven out of every 100 000 people [6]. It is a rapidly progressing disease with a poor prognosis. Despite numerous effective measures taken in recent years to address ARDS, the mortality rate for patients with ARDS remains above 25% [7, 8, 9]. Therefore, the early identification and diagnosis of patients with sepsis and those at risk of ARDS are critical to improving patient prognosis and reducing mortality.

LncRNA is a noncoding RNA with a length of over 200 nucleotides [10]. It plays a crucial role in regulating cell activities and determining cell fate, thereby ensuring the normal process of organism reproduction, growth, development, and other life activities [11]. There is increasing evidence that lncRNA is related to the occurrence and development of many major diseases [12]. Consequently, lncRNA is becoming promising disease markers and drug targets, succeeding protein molecules [13]. LncRNA has also been demonstrated to be associated with sepsis and ARDS in sepsis. For instance, the downregulation of LncRNA NEAT1 may improve the inflammatory response and cell cycle progression of sepsis‐induced ARDS [14]. LncRNA MEG3 is abnormally expressed in ARDS patients and positively correlates with disease severity [15]. HOXA antisense RNA 2 (HOXA‐AS2) is a lncRNA of length 1048, located between the HOXA3 and HOXA4 genes [16]. Study found that upregulation of HOXA‐AS2 can reverse acute kidney injury caused by sepsis [17]. HOXA‐AS2 can alleviate lung inflammation caused by chronic intermittent hypoxia. Overexpression of HOXA‐AS2 mitigates lung injury in chronic intermittent hypoxia rats by inhibiting apoptosis, improving hypoxemia, and altering the level of inflammatory factors [18]. HOXA‐AS2 is downregulated in patients with chronic obstructive pulmonary disease (COPD) and is associated with endothelial cell proliferation [19]. HOXA‐AS2 has also been shown to inhibit endothelial cell inflammation [20]. Pulmonary capillary endothelial cells are not just the target cells of inflammation in ARDS but also important effector cells [21]. The breakdown of pulmonary capillary endothelial cell barrier plays a pivotal role in the occurrence and development of ARDS [22]. However, whether HOXA‐AS2 is associated with the development of ARDS in sepsis remains unclear. Whether HOXA‐AS2 is a valuable predictive biomarker of ARDS in sepsis requires further investigation.

Based on previous studies, we hypothesize that HOXA‐AS2 plays a role in the occurrence and development of sepsis and sepsis‐related ARDS. This study aims to explore the mechanism of HOXA‐AS2 involved in sepsis‐related ARDS and the predictive value of this molecule for ARDS and 28‐day mortality. This may provide valuable biomarkers for the diagnosis, treatment, and prognosis of sepsis and ARDS.

## 2 Materials and Methods

### 2.1 Participants

From July 2019 to August 2020, 122 patients with sepsis were enrolled in Wuxi Branch of Zhongda Hospital Southeast University hospital. The enrollment criteria were as follows: (a) diagnosed as sepsis patients [23]; (b) age over 18 years; (c) admission to hospital within 24 h; (d) no immunosuppressive therapy in the last 3 months; (e) no complications from other fatal diseases; and (f) no cancer or malignant tumors. Patients with sepsis did not meet the diagnostic criteria for ARDS before enrollment. Additionally, we recruited 101 healthy volunteers without sepsis to serve as a control group. Both groups were matched for age and gender. The criteria for the control group included (a) the physical examination result is normal; (b) no history of cancer or sepsis; and (c) no immunosuppressive drugs have been used recently.

Our study received approval from the Institutional Review Board of Zhongda Hospital Southeast University's Wuxi Branch. All subjects also provided written informed consent.

### 2.2 Specimen Collection

Upon admission, we recorded the patient's baseline clinical features in detail. We diagnosed the severity of sepsis in the patient using the SOFA score and APACHE II score within 24 h. We collected blood samples from both the control group and sepsis patients on their respective enrollment and admission days. We stored the separated serum at −80°C.

### 2.3 RNA Extraction and Real‐Time Quantitative Reverse Transcription PCR (RT‐qPCR)

First, we isolated the serum total RNA using the TRIZOL regent. We then determined the RNA purity and concentration using the NanoDrop 1000 spectrophotometer. Second, we synthesized cDNA from 2 μg of RNA. Finally, we performed amplification reactions after mixing cDNA, primers, RNase Free H_2_O, and SYBR Green Quantitative RT‐qPCR Kit reagent well. GAPDH is the reference gene. The relative expression of HOXA‐AS2 was calculated by 2^−ΔΔCt^ method.

### 2.4 ARDS Assessment

After admission, patients with sepsis were closely monitored. ARDS was diagnosed using the Berlin definition [24], which includes (a) onset within 7 days of clinical injury; (b) decreased translucency in both lungs that cannot be fully explained by lobar atelectasis, pleural effusion, or nodules; (c) edema, respiratory failure (not fully explained by cardiac failure or fluid overload); (d) oxygenation (partial pressure of arterial oxygen/fraction of inspired oxygen (PaO_2_/FIO_2_) ≤ 300 mmHg with positive end‐expiratory pressure (PEEP) ≥ 5‐cm H_2_O).

### 2.5 Cell Culture, Treatment, and Transfected

Human pulmonary microvascular endothelial cells (HPMECs) were purchased from Pricella Biotech (Wuhan, China). The cells were cultured in a complete culture medium (Pricella, Wuhan, China), under conditions of 37°C and 5% CO2. To construct an in vitro ARDS cell model, we treated HPMECs with 100ng/mL lipopolysaccharide (LPS) for 24 h.

Overexpressing plasmids of HOXA‐AS2 (oe‐HOXA‐AS2) and the negative control (oe‐NC) were synthesized by GenePharma (Shanghai, China). When HPMECs are in the logarithmic growth phase, we transfected the oe‐HOXA‐AS2 and oe‐NC plasmids into HPMECs using Lipo6000 (Beyotime, Shanghai, China).

### 2.6 Cell Viability Assay

We inoculated HPMEC cells into 96‐well plates (5 × 10^3^ cells per well). After incubation, 10‐μL CCK‐8 solution was added to each well. After 2 h of incubation, we measured the absorbance at 450 nm using a microplate reader.

### 2.7 Cell Apoptosis Assay

The Annexin V‐APC/Cyannine7/PI Apoptosis Kit (Elabscience, Wuhan, China) was used for apoptosis detection. HPMECs were first cleaned with PBS buffer. After centrifugation, the supernatant was discarded, and the cells were resuspended in 500 μL of 1 × Annexin V Binding Buffer. Subsequently, 5 μL of Annexin V‐APC/Cyannine7 Regent and PI Regent were added to the suspension. The cell samples were then incubated at room temperature in the dark for 20 min. Following the incubation, apoptotic cells were detected using flow cytometry.

### 2.8 Enzyme‐Linked Immunosorbent Assay (ELISA)

The concentrations of IL‐6, TNF‐α, IL‐1β, and IL‐8 were measured using commercial Human IL‐6, TNF‐α, IL‐1β, and IL‐8 ELISA Kits (Solarbio, Beijing, China). We strictly adhered to the instructions provided with the kit.

### 2.9 Statistical Analysis

SPSS 23.0 software (IBM) and GraphPad Prism 9.0 software were used for statistical analysis and charting. The data were presented as mean ± standard deviation (SD), mean with interquartile range (IQR), and number (percentage). The student's *t*‐test and the Mann–Whitney *U* test were used for comparison of differences among variables, and the Spearman's rank correlation test was used for correlation analysis. The diagnostic value of HOXA‐AS2 in sepsis and ARDS was analyzed using receiver operating characteristic (ROC) curve. The Kaplan–Meier curve was used to describe the relationship between HOXA‐AS2 levels and cumulative mortality in sepsis patients. The logistic regression analysis and COX regression analysis were used to analyze the risk factors affecting ARDS and 28‐day mortality. *p* < 0.05 was considered statistically significant.

## 3 Results

### 3.1 Clinical Characteristics of Sepsis Patients

In this study, 122 sepsis patients were enrolled. The average age was 54.78 ± 10.06 years, with 58.20% being male. COPD was found in 20.49% of sepsis patients, cardiomyopathy in 39.34%, chronic renal failure in 16.39%, and cirrhosis in 21.31%. In terms of infection types, 45 (36.89%) of the patients had abdominal infections, 25 (20.49%) had respiratory infections, 23 (18.85%) had skin and soft tissue infections, 14 (11.48%) had bloodstream infections, 7 (5.74%) had central nervous system infections, and 8 (6.56%) had other types of infections. Additional details are listed in Table 1.

**TABLE 1:** Comparison of basic clinical information of patients with sepsis.

Parameters	Sepsis patients (*n* = 122)	Control (*n* = 101)	*p*
Age (y), mean ± SD	54.78 ± 10.06	54.69 ± 5.67	0.939
Male, no. (%)	71 (58.20)	51 (50.50)	0.225
BMI (kg/m^2^), mean ± SD	22.63 ± 3.88	23.14 ± 2.82	0.274
Smoke, no. (%)	47 (38.52)	42 (41.58)	0.681
**Complications, no. (%)**			
COPD	25 (20.49)	—	—
Cardiomyopathy	48 (39.34)	—	—
Chronic kidney failure	20 (16.39)	—	—
Cirrhosis	26 (21.31)	—	—
**Primary infection site, no. (%)**		—	—
Abdominal infection	45 (36.89)	—	—
Respiratory infection	25 (20.49)	—	—
Skin and soft tissue infection	23 (18.85)	—	—
Bloodstream infection	14 (11.48)	—	—
CNS infection	7 (5.74)	—	—
Other infection	8 (6.56)	—	—
**Primary organism,** no. (%)			
G^−^ bacteria	65 (53.28)	—	—
G^+^ bacteria	23 (18.85)	—	—
Anaerobe	14 (11.48)	—	—
Fungus	8 (6.56)	—	—
Mycoplasmas	3 (2.46)	—	—
Total culture negative	19 (15.57)	—	—
**Biochemical indexes, median (IQR)**			
Scr (mg/dL)	1.89 (1.32–2.48)	0.99 (0.88, 1.09)	< 0.001
Albumin, (g/L)	23.29 (23.74–30.44)	41.33 (38.79, 44.32)	< 0.001
WBC (×10^9^/L)	21.25 (14.86–25.61)	7.27 (6.44, 8.00)	< 0.001
CRP (mg/L)	72.21 (44.03–147.07)	6.11 (4.73, 7.62)	< 0.001
**Disease severity, median (IQR)**			
APAHCE II score	15.5 (12.00–18.00)	—	—
SOFA score	7.5 (5.0–10.0)	—	—

Abbreviations: APACHE, acute physiology and chronic health evaluation; BMI, body mass index; CNS, central nervous system; COPD, chronic obstructive pulmonary disease; CRP, C‐reactive protein; Scr, serum creatinine; SOFA, sequential organ failure assessment; WBC, white blood cells.

### 3.2 HOXA‐AS2 Distinguishes Sepsis Patients From Controls

**FIGURE 1:**
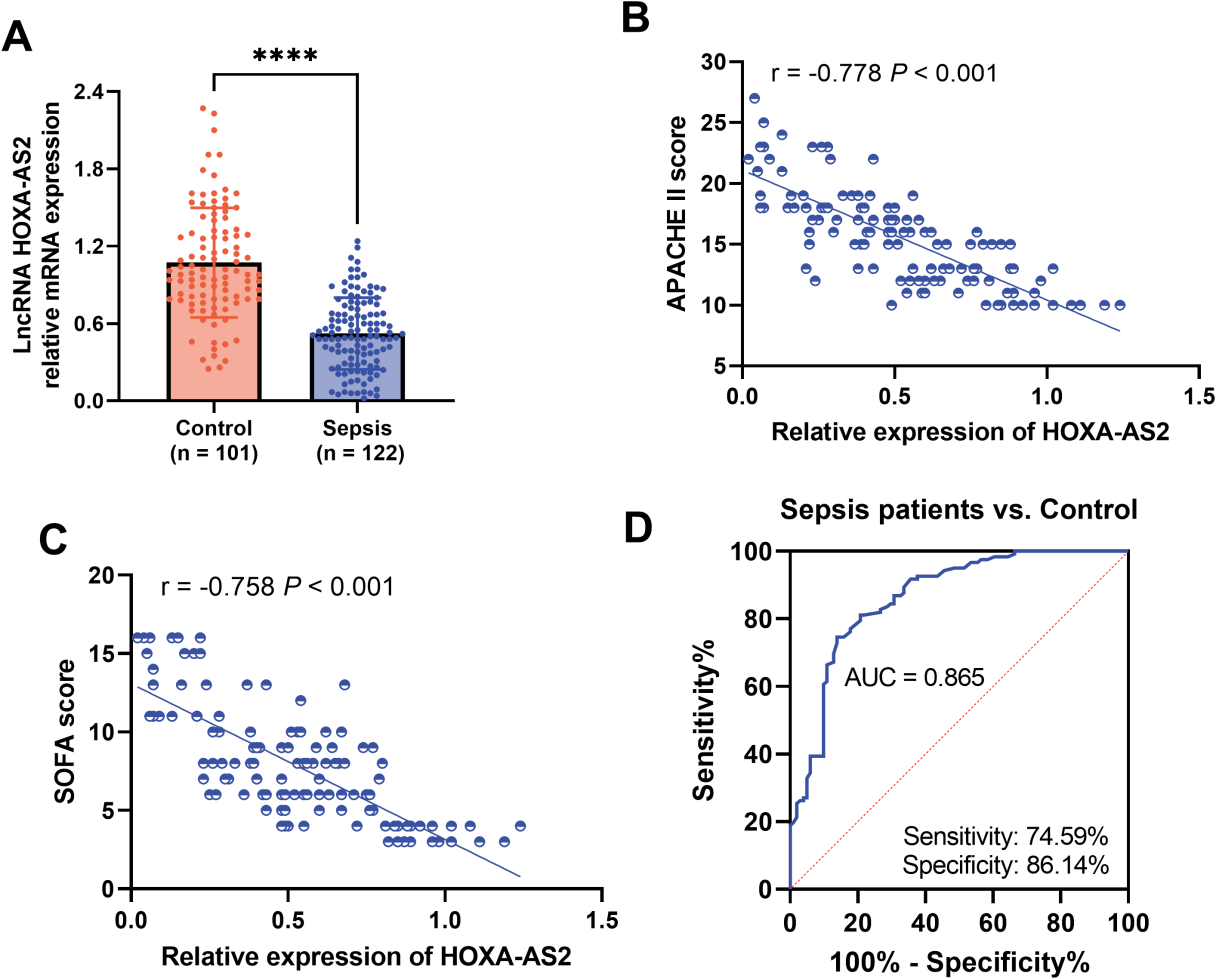
HOXA‐AS2 is a diagnostic marker for sepsis. (A) The level of HOXA‐AS2 was determined by RT‐qPCR. (B–C) *. APACHE* II score and SOFA score were negatively correlated with HOXA‐AS2. (D) The performance of HOXA‐AS2 in distinguishing sepsis patients from healthy controls was analyzed by receiver operating characteristic (ROC) curve. ****p < 0.0001.

Compared to controls, HOXA‐AS2 levels were significantly lower in sepsis patients (*p* < 0.0001, Figure 1A). HOXA‐AS2 levels had a negatively correlation with the APACHE II score (*r* = −0.778, *p* < 0.001, Figure 1B). Similarly, there was a negative correlation between HOXA‐AS2 and the SOFA score (*r* = −0.758, *p* < 0.001, Figure 1C). Furthermore, HOXA‐AS2 could differentiate sepsis patients from controls (AUC = 0.865, sensitivity = 74.59%, specificity = 86.14%, Figure 1D).

### 3.3 Clinical Characteristics of ARDS Sepsis Patients and Non‐ARDS Sepsis Patients

During the 28‐day follow‐up, 32 sepsis patients developed ARDS. Compared to sepsis patients without ARDS, sepsis with ARDS had higher smoking rates (*p* = 0.022). The proportion of patients with COPD (*p* = 0.023) and respiratory infections (*p* = 0.001) was also higher in the ARDS group (Table 2). Additionally, the CRP (*p* = 0.022), SOFA score (*p* = 0.000), and APACHE II score (*p* = 0.003) were significantly elevated in sepsis patients with ARDS (Table 2).

**TABLE 2:** Comparison of basic clinical information of patients with non‐ARSD sepsis and ARDS sepsis.

Parameters	Non‐ARDS sepsis patients (*n* = 90)	ARDS sepsis patients (*n* = 32)	*p*
Age (y), mean ± SD	55.69 ± 8.99	52.22 ± 12.41	0.094
Male, no. (%)	53 (58.89)	18 (56.25)	0.795
BMI (kg/m^2^), mean ± SD	22.34 ± 3.97	23.46 ± 3.56	0.161
Smoke, no. (%)	34 (37.78)	16 (50.00)	**0.022**
**Complications, no. (%)**			
COPD	14 (15.56)	11 (34.38)	**0.023**
Cardiomyopathy	35 (38.89)	13 (40.63)	0.863
Chronic kidney failure	13 (14.44)	77 (21.88)	0.329
Cirrhosis	20 (22.22)	6 (18.75)	0.680
**Primary infection site, no. (%)**			
Abdominal infection	38 (42.22)	7 (21.88)	0.054
Respiratory infection	11 (12.22)	14 (43.75)	**0.001**
Skin and soft tissue infection	17 (18.89)	6 (18.75)	0.986
Bloodstream infection	12 (13.33)	2 (6.25)	0.352
CNS infection	5 (5.56)	2 (6.25)	0.885
Other infection	7 (7.78)	1 (3.13)	0.679
**Primary organism,** no. (%)			
G^−^ bacteria	47 (52.22)	18 (56.25)	0.837
G^+^ bacteria	17 (18.89)	6 (18.75)	0.986
Anaerobe	9 (10.00)	5 (15.63)	0.391
Fungus	6 (6.67)	2 (6.25)	0.935
Mycoplasmas	2 (2.22)	1 (3.13)	0.777
Total culture negative	13 (14.44)	6 (18.75)	0.564
**Biochemical indexes, median (IQR)**			
Scr (mg/dL)	1.89 (1.32–2.44)	1.93 (1.32–2.68)	0.896
Albumin, (g/L)	27.29 (24.01–31.10)	26.70 (22.27–30.13)	0.511
WBC (×10^9^/L)	19.65 (14.35–25.40)	23.52 (19.83–26.52)	0.076
CRP (mg/L)	67.72 (43.05–126.79)	131.48 (49.71–186.18)	**0.022**
**Disease severity, median (IQR)**			
APAHCE II score	15.00 (11.00–17.00)	17.00 (15.00–19.00)	**0.003**
SOFA score	6.00 (4.00–8.25)	13.00 (8.00–15.00)	**0.000**

Abbreviations: APACHE, acute physiology and chronic health evaluation; BMI, body mass index; CNS, central nervous system; COPD, chronic obstructive pulmonary disease; CRP, C‐reactive protein; Scr, serum creatinine; SOFA, sequential organ failure assessment; WBC, white blood cells.

### 3.4 HOXA‐AS2 Predicts ARDS Risk in Sepsis Patients

**FIGURE 2:**
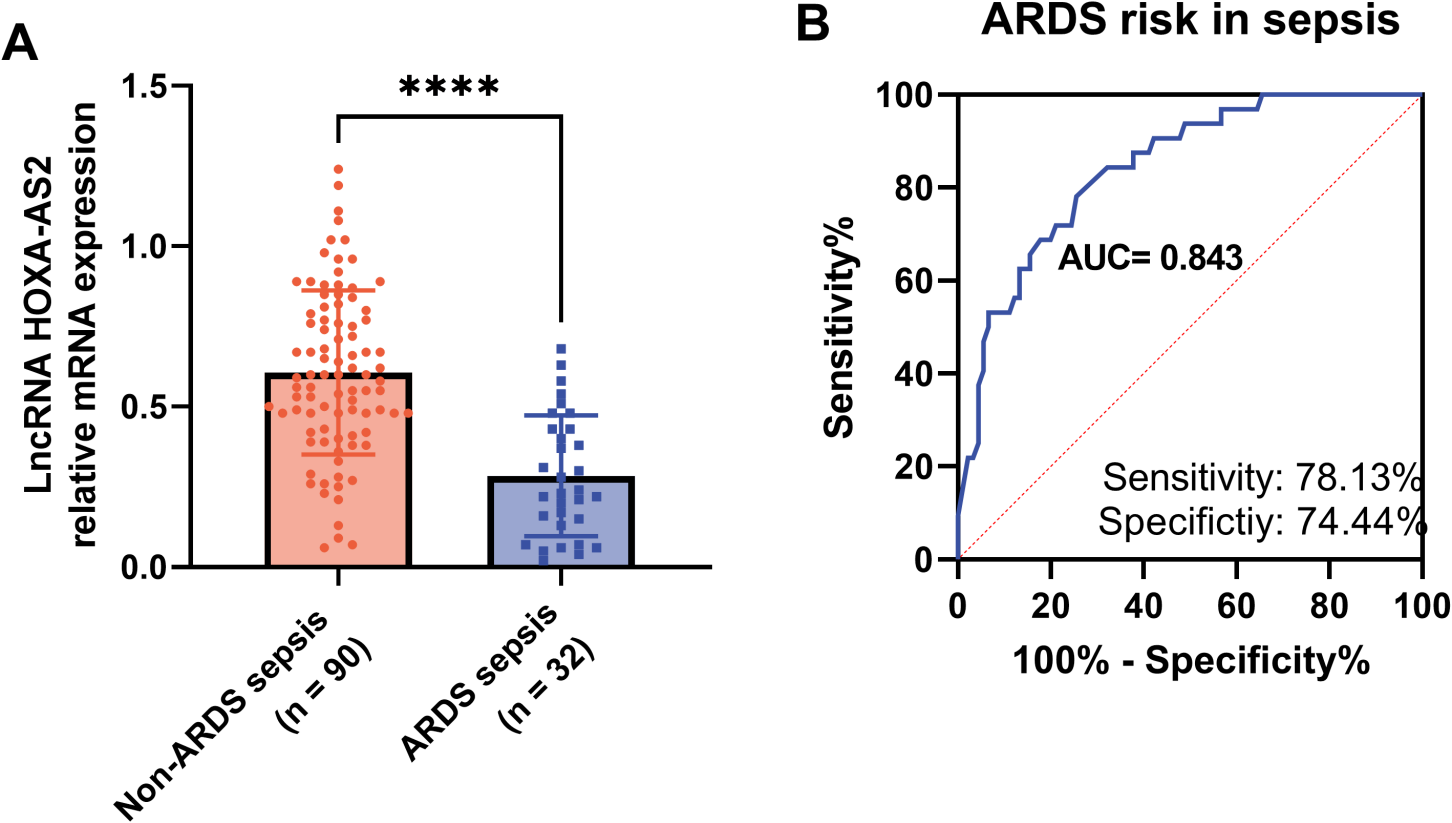
In patients with sepsis, HOXA‐AS2 can be used as a diagnostic marker for acute respiratory distress syndrome (ARDS). (A) The level of HOXA‐AS2 was determined by RT‐qPCR. (B) The performance of HOXA‐AS2 in distinguishing ARDS patients from non‐ARDS patients was analyzed by receiver operating characteristic (ROC) curve. ****p < 0.0001.

Compared to non‐ARDS sepsis patients, HOXA‐AS2 levels were significantly decreased in ARDS sepsis patients (*p* < 0.0001, Figure 2A). HOXA‐AS2 can differentiate ARDS sepsis patients from non‐ARDS sepsis patients (AUC = 0.843, sensitivity = 78.13%, specificity = 74.44%. Figure 2B).

As is shown in Table 3, we used logistic regression analysis to examine the clinical characteristics and the level of HOXA‐AS2 in ARDS sepsis patients and non‐ARDS sepsis patients, in order to analyze risk factors for the development of ARDS. The results indicated that HOXA‐AS2 (*p* = 0.002), COPD (*p* = 0.029), primary infection site (respiratory vs. others) (*p* = 0.005), and SOFA score (*p* = 0.004) were independent risk factors for ARDS.

**TABLE 3:** Analysis of risk factors of ARDS in patients with sepsis.

Parameters	Univariate logistic regression			Multivariate logistic regression		
	OR	95%CI	*p*	OR	95%CI	*p*
Age	0.749	0.317–1.770	0.510			
Gender	0.898	0.397–2.028	0.795			
BMI (kg/m^2^)	0.807	0.360–1.811	0.604			
Smoke	2.571	1.127–5.865	**0.025**	0.434	0.154–1.222	0.114
COPD	2.844	1.127–7.177	**0.027**	0.227	0.060–0.860	**0.029**
Cardiomyopathy	1.075	0.472–2.448	0.863			
Chronic kidney failure	1.658	0.596–4.616	0.333			
Cirrhosis	1.238	0.448–3.424	0.681			
Primary infection site (respiratory vs. others)	3.889	1.594–9.486	**0.003**	0.177	0.052–0.600	**0.005**
Primary infection organism						
G^−^ vs. others	1.176	0.522–2.649	0.695			
G^+^ vs. others	1.009	0.359–2.834	0.986			
Anaerobes/fungi/mycoplasmas vs. others	1.431	0.549–3.733	0.463			
Scr	1.344	0.597–3.026	0.475			
Albumin	1.286	0.571–2.895	0.544			
WBC	1.996	0.863–4.616	0.106			
CRP	2.387	1.041–5.474	**0.040**	0.548	0.190–1.578	0.265
APAHCE II score	2.386	1.031–5.526	**0.042**	0.394	0.105–1.478	0.167
SOFA score	4.297	1.741–10.608	**0.004**	0.152	0.042–0.554	**0.004**
HOXA‐AS2	8.100	2.854–22.992	0.000	0.100	0.023–0.426	**0.002**

Abbreviations: APACHE, acute physiology and chronic health evaluation; BMI, body mass index; CNS, central nervous system; COPD, chronic obstructive pulmonary disease; CRP, C‐reactive protein; Scr, serum creatinine; SOFA, sequential organ failure assessment; WBC, white blood cells.

### 3.5 lncRNA HOXA‐AS2 Can Predict 28‐Day Mortality in Sepsis Patients

**FIGURE 3:**
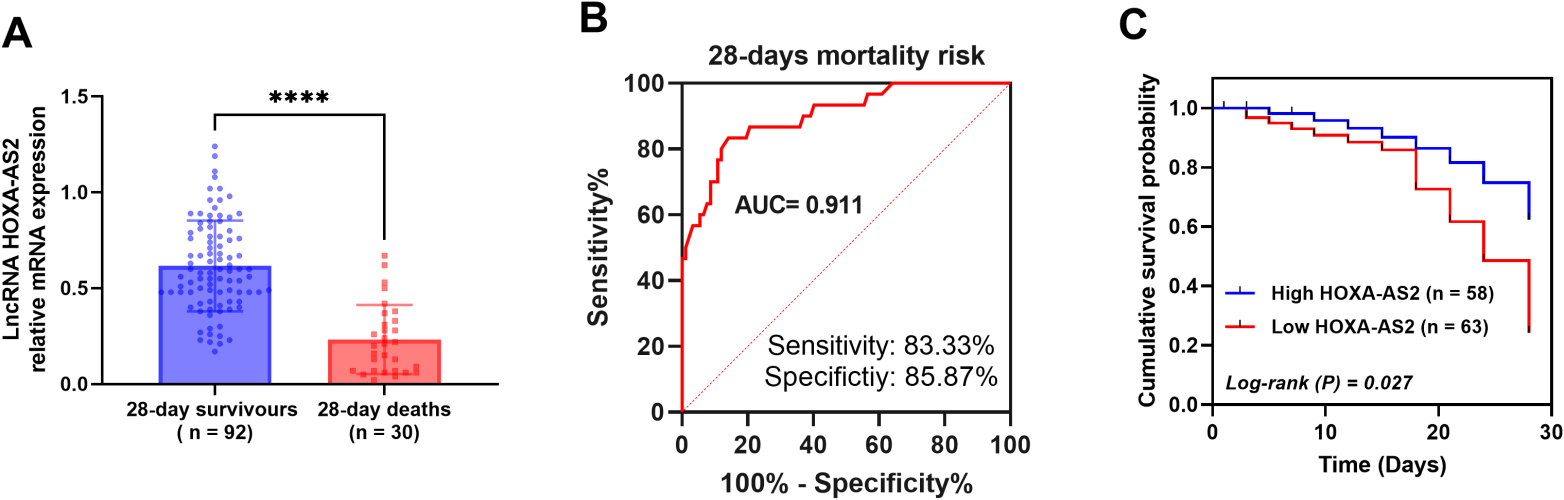
Serum HOXA‐AS2 significantly predicts 28‐day mortality in sepsis patients. (A) The level of HOXA‐AS2 was determined by RT‐qPCR. (B) The predictive value of HOXA‐AS2 in 28‐day mortality risk was analyzed by receiver operating characteristic (ROC) curve. (C) The relationship between HOXA‐AS2 levels and the survival rate of patients with sepsis was analyzed by the Kaplan–Meier curve. ****p < 0.0001.

During the 28‐day follow‐up period, there were 30 (24.59%) deaths and 92 (75.41%) survivors. Compared to survivors, HOXA‐AS2 levels were significantly decreased in those who died within 28 day (*p* < 0.0001, Figure 3A). The ROC curve suggests that the relative expression level of HOXA‐AS2 can predict mortality risk in sepsis patients (AUC = 0.911, sensitivity = 83.33%, specificity = 85.87%. Figure 3B). Moreover, sepsis patients with lower HOXA‐AS2 expression have a higher mortality rate (log‐rank *p* = 0.027, Figure 3C). Multivariate COX regression analysis shows that HOXA‐AS2 (*p* = 0.005, HR = 5.380), SOFA score (*p* = 0.040, HR = 3.960) and APACHE II score (*p* = 0.013, HR = 5.380) are independent risk factors for death in sepsis patients (Table 4).

**TABLE 4:** Cox regression analysis of independent risk factors for 28‐day mortality in patients with sepsis.

Parameters	Univariate Cox			Multivariate Cox		
	HR	95%CI	*p*	HR	95%CI	*p*
Age	1.002	0.483–2.079	0.996			
Gender	0.809	0.382–1.715	0.580			
BMI (kg/m^2^)	0.956	0.461–1.981	0.903			
Smoke	2.589	1.136–5.901	**0.024**	1.783	0.669–4.752	0.247
COPD	2.313	1.110–4.819	**0.025**	1.002	0.333–3.016	0.997
Cardiomyopathy	0.287	0.305–1.420	0.658			
Chronic kidney failure	0.373	0.088–1.578	0.180			
Cirrhosis	0.811	0.324–2.027	0.654			
Primary infection site (respiratory vs. others)	2.209	1.056–4.623	**0.035**	2.996	0.856–4.338	0.113
Primary infection organism						
G^−^ vs. others	0.899	0.431–1.877	0.777			
G^+^ vs. others	0.743	0.298–1.850	0.523			
Anaerobes/fungi/mycoplasmas vs. others	0.799	0.324–1.967	0.625			
Scr	2.303	1.047–5.065	**0.038**	1.927	0.856–4.338	0.113
Albumin	2.039	0.902–4.608	**0.087**	1.090	0.440–5.697	0.852
WBC	2.323	0.942–5.725	**0.067**	1.352	0.500–3.659	0.552
CRP	3.483	1.417–8.563	**0.007**	2.588	0.831–8.060	0.101
APAHCE II score	2.370	1.048–5.358	**0.038**	4.010	1.334–12.056	**0.013**
SOFA score	5.571	1.672–18.583	**0.005**	3.960	1.062–14.762	**0.040**
HOXA‐AS2	2.587	1.146–5.841	**0.022**	5.380	1.499–19.308	**0.010**

Abbreviations: APACHE, acute physiology and chronic health evaluation; BMI, body mass index; CNS, central nervous system; COPD, chronic obstructive pulmonary disease; CRP, C‐reactive protein; Scr, serum creatinine; SOFA, sequential organ failure assessment; WBC, white blood cells.

### 3.6 Overexpression of HOXA‐AS2 Alleviates LPS‐Induced Damage to HPMECs

**FIGURE 4:**
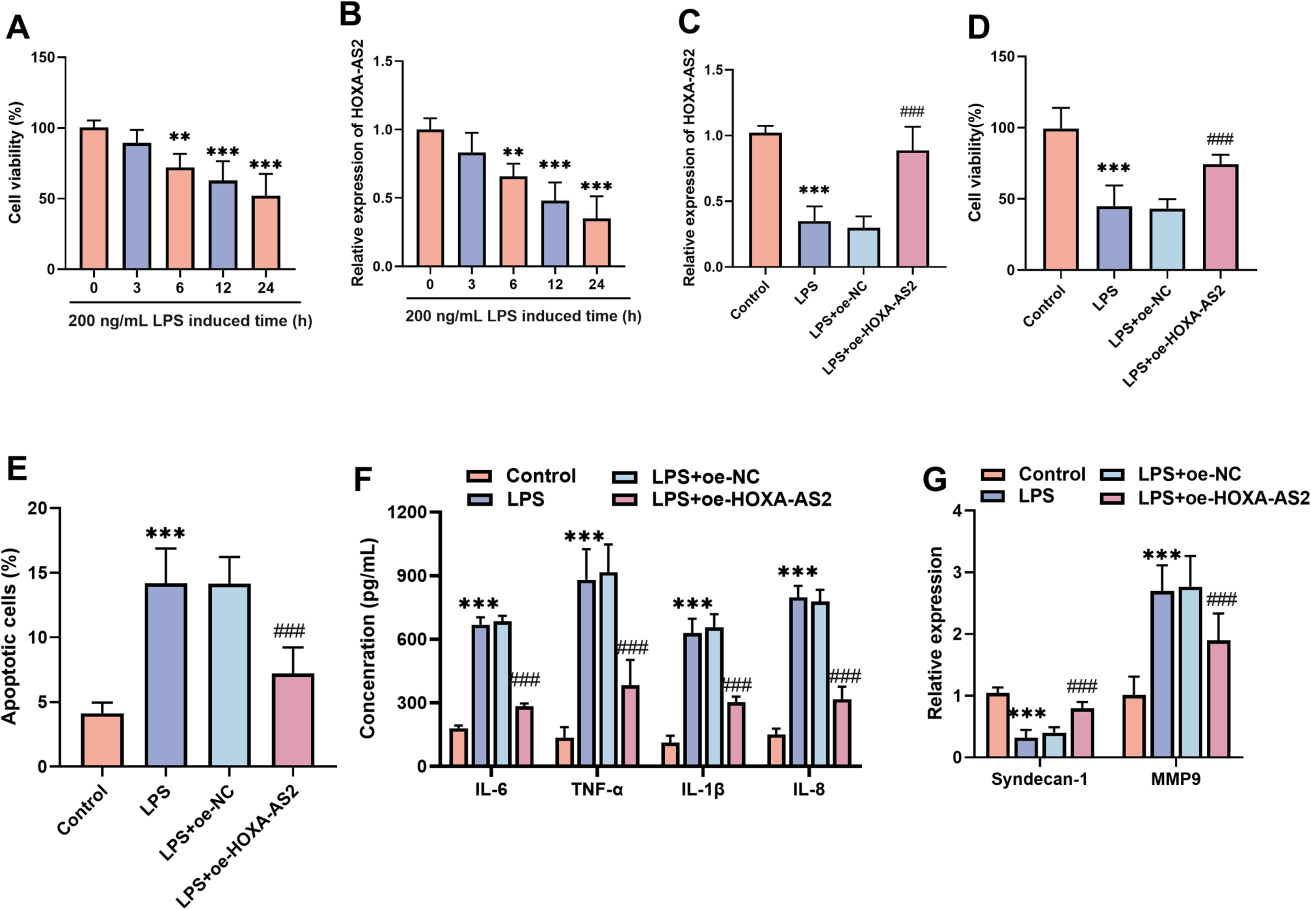
HOXA‐AS2 restrained LPS‐induced damage to human pulmonary microvascular endothelial cells (HPMECs). (A) Cell viability of HPMECs after LPS treatment. CCK‐8 method was used to determine cell viability. (B) The level of HOXA‐AS2 after LPS treatment. The level of HOXA‐AS2 was determined by RT‐qPCR. (C) The effect of transfection with overexpression plasmids on HOXA‐AS2. (D) CCK‐8 method was used to determine cell viability. (E) Apoptosis kits and flow cytometry were used to measure apoptosis rate. (F) The levels of inflammatory factors were measured by ELISA assay. (G) the mRNA levels of syndecan‐1 and MMP9 were measured by the RT‐qPCR. ***p < 0.001 vs. Control; ###*p* < 0.001 vs. LPS + oe‐NC.

LPS can inhibit cell growth, and cell viability decreases as treatment time extends (*p* < 0.01, Figure 4A). This validates the successful construction of the ARDS cell model in vitro. In vitro, LPS significantly decreased the level of HOXA‐AS2 (*p* < 0.01, Figure 4B). However, this downregulation is usually reversed by overexpressing HOXA‐AS2 (*p* < 0.001, Figure 4C). As shown in Figure 4, LPS can inhibit the proliferation of HPMECs, promote apoptosis, and increase the level of inflammatory factors (IL‐6, TNF‐α, IL‐1β, and IL‐8). However, overexpression of HOXA‐AS2 can reverse these results (*p* < 0.001, Figure 4D–F). We also measured the levels of Syndecan‐1 and MMP9 under different conditions, LPS decreased and increased the levels of Syndecan‐1 and MMP9, respectively. After upregulating HOXA‐AS2, we observed the opposite result (*p* < 0.001, Figure 4G).

## 4 Discussion

Sepsis is a life‐threatening organ dysfunction caused by a dysfunctional response to infection [25]. Despite the availability of effective antibiotics and established life‐supporting treatments, sepsis continues to have a high mortality rate [26]. ARDS is a diffuse lung injury and acute respiratory failure due to various causes, characterized by damage to alveolar epithelial cells and pulmonary capillary endothelial cells [27]. Research indicates that sepsis associated ARDS has a higher disease severity and mortality rate than non‐sepsis associated ARDS [28]. Sepsis‐associated ARDS accounts for about 30% of sepsis patients, and the fatality rate reaches 30%–40% [29]. Sepsis associated ARDS is a heterogeneous clinical syndrome with a complex pathogenesis, and our ability to predict its development and prognosis is still limited. Biomarkers can help identify sepsis patients at an early stage, predict the risk of ARDS, and stratify patients at risk and reveal prognosis. While there are still no targeted drug treatments for ARDS, biomarkers can help deepen our understanding of the pathophysiology and identify potential new therapeutic targets and strategies.

In our study, we found that the level of HOXA‐AS2 was significantly reduced in sepsis patients compared to non‐sepsis patients. This finding aligns with Huifeng Wu's study [17], which also reported decreased levels of HOXA‐AS2 in sepsis patients. Moreover, our study found that HOXA‐AS2 is not only a diagnostic biomarker for sepsis but also can predicts the onset of ARDS in sepsis patients. Levels of HOXA‐AS2 are lower in sepsis patients who develop ARDS compared to those who do not. Our multivariate logistic regression analysis further indicates that HOXA‐AS2 is an independent risk factor for ARDS in sepsis patients. Research has shown that the occurrence of ARDS triggers macrophages to release pro‐inflammatory cytokines [30]. This surge in pro‐inflammatory cytokines causes damage to alveolar epithelial cells and capillary endothelium [31]. HOXA‐AS2 has been shown to play an inhibitory role in endothelial cell inflammation [20, 32]. Therefore, decreased HOXA‐AS2 levels could be a significant cause of ARDS in sepsis patients. HOXA‐AS2 cannot only serve as a diagnostic marker for patients with sepsis but also act as a risk factor to indicate the risk of ARDS.

Prognosis plays a crucial role in medicine as it helps predict the course and outcome of a disease. Evaluating prognosis assists doctors in devising a treatment plan to better manage disease progression and improve patients' quality of life. Therefore, we also evaluated the relationship between HOXA‐AS2 levels and the prognosis of sepsis patients. After a 28‐day follow up, we found that HOXA‐AS2 levels were significantly lower in patients who died. This led us to hypothesize that a decreased HOXA‐AS2 level correlates with a higher mortality rate in sepsis patients. To further validate our hypothesis, we analyzed the correlation between HOXA‐AS2 levels and SOFA score and APACHE II score. The SOFA score and APACHE II score are commonly used to assess the severity of sepsis and to predict the prognosis of sepsis patients. Higher scores indicate more severe organ damage and a worse prognosis. Our findings showed that HOXA‐AS2 levels negatively correlated with both SOFA and APACHE II scores, suggesting that lower HOXA‐AS2 levels are associated with more severe sepsis. A multivariate logistic regression analysis further confirmed that a reduced HOXA‐AS2 level is an independent risk factor for death in sepsis patients. Therefore, our results indicate that the level of HOXA‐AS2 is closely related to the mortality rate of patients with sepsis. Clinically, it may be possible to assist in screening patients with a high risk of death by detecting the level of HOXA‐AS2.

To further explore the mechanism of HOXA‐AS2 involved in the development of ARDS, we constructed a sepsis‐associated ARDS cell model by treating HPMEC cells with LPS. LPS is a component of the outer membrane of Gram‐negative bacteria that can trigger dysregulated host responses and inflammation in sepsis and ARDS [33]. In our study, LPS reduced the cell viability of HPMEC, accelerated apoptosis, and encouraged the secretion of pro‐inflammatory factors such as IL‐6, TNF‐α, IL‐1β, and IL‐8. These effects were reversed by overexpressing HOXA‐AS2, suggesting that HOXA‐AS2 inhibits the progression of sepsis‐induced ARDS by suppressing the inflammatory response. Increased endothelial permeability is another major characteristic of ARDS. Studies have demonstrated that elevated vascular endothelial permeability can cause pulmonary edema, eventually leading to respiratory failure. The degradation of the endothelial glycocalyx, a layer of polyglycoprotein complexes that cover endothelial cells with an average thickness of 0.1–2 um, is a significant factor in increasing vascular endothelial permeability [34]. Degradation of the endothelial glycocalyx was found to occur in sepsis and sepsis‐induced ARDS [35]. The endothelial glycocalyx is associated with vascular homeostasis and organ dysfunction in sepsis [36]. Syndecan‐1 is a core protein comprising the endothelial glycocalyx, and it is an important structural component of the endothelial glycocalyx [37]. MMP‐9, which can directly degrade Syndecan‐1, is closely associated with the degradation of the endothelial glycocalyx [38]. In our study, in addition to its role in inflammatory events in ARDS, we found that HOXA‐AS2 is associated with endothelial glycocalyx degradation. Overexpression of HOXA‐AS2 increased Syndecan‐1 levels and decreased MMP‐9 levels, alleviating LPS‐induced endothelial glycocalyx degradation. Therefore, our results suggest that the decrease in HOXA‐AS2 levels may accelerate the occurrence and progression of ARDS by promoting inflammation and glycocalyx degradation in HPMEC cells.

To sum up, our research confirmed that HOXA‐AS2 cannot only serve as a diagnostic marker for patients with sepsis but also predict the risk of ARDS and death in patients with sepsis. The decreased level of HOXA‐AS2 increases the probability of ARDS and death in patients with sepsis. HOXA‐AS2 may partially delay the occurrence and development of ARDS by inhibiting the inflammation of pulmonary microvascular endothelial cells and the degradation of glycocalyx.

## Ethics Statement

The study protocol was approved by The Ethics Committee of Wuxi Branch of Zhongda Hospital Southeast University and followed the principles outlined in the Declaration of Helsinki.

## Consent

In addition, informed consent has been obtained from the participants involved.

## Conflicts of Interest

The authors declare no conflicts of interest.

## Data Availability

The datasets used and/or analyzed during the current study are available from the corresponding author on reasonable request.
